# Modulation of u-PA, MMPs and their inhibitors by a novel nutrient mixture in pediatric human sarcoma cell lines

**DOI:** 10.3892/ijo.2013.2031

**Published:** 2013-07-23

**Authors:** M. WAHEED ROOMI, TATIANA KALINOVSKY, ALEKSANDRA NIEDZWIECKI, MATTHIAS RATH

**Affiliations:** Dr Rath Research Institute, Santa Clara, CA, USA

**Keywords:** osteosarcoma MNNG-HOS and U-2OS, rhabdomyosarcoma RD, urokinase plasminogen activator, matrix metalloproteinase-2, matrix metalloproteinase-9, tissue inhibitor of metalloproteinase-2, PMA, nutrient mixture

## Abstract

Pediatric sarcomas are highly aggressive tumors that are characterized by high levels of matrix metalloproteinase (MMP)-2 and -9 secretions that degrade the ECM and basement membrane, allowing cancer cells to spread to distal organs. Proteases play a key role in tumor cell invasion and metastasis by digesting the basement membrane and ECM components. Strong clinical and experimental evidence demonstrates association of elevated levels of u-PA and MMPs with cancer progression, metastasis and shortened patient survival. MMP activities are regulated by specific tissue inhibitors of metalloproteinases (TIMPs). Our main objective was to study the effect of a nutrient mixture (NM) on activity of u-PA, MMPs and TIMPs in various human pediatric sarcomas. Human osteosarcoma MNNG-HOS, osteosarcoma U-2OS and rhabdomyosarcoma RD cell lines (ATCC) were cultured in their respective media and treated at confluence with NM at 0, 50, 100, 250, 500 and 1,000 *μ* g/ml. Analysis of u-PA activity was carried out by fibrin zymography, MMPs by gelatinase zymography and TIMPs by reverse zymography. All sarcoma cell lines studied expressed u-PA, which was inhibited by NM in a dose-dependent manner. On gelatinase zymography, osteosarcoma MNNG-HOS showed a band corresponding to MMP-2 and induction of MMP-9 with PMA (100 ng/ml) treatment. U-2OS osteosarcoma cells showed strong bands corresponding to inactive MMP-2 and MMP-9 and faint bands corresponding to active MMP-2 and MMP-9 dimer; PMA treatment enhanced MMP-9 and MMP-9 dimer activity. Rhabdomyosarcoma showed MMP-2 and faint MMP-9 bands; PMA treatment enhanced MMP-9 expression. NM inhibited their expression in a dose-dependent manner. Activity of TIMPs was upregulated by NM in all cancer cell lines in a dose-dependent manner. Analysis revealed a positive correlation between u-PA and MMPs and a negative correlation between u-PA/MMPs and TIMPs. These findings suggest the therapeutic potential of NM in treatment of pediatric sarcomas.

## Introduction

Osteosarcoma, the most common bone cancer in children, accounting for about 5% of all childhood cancers, usually presents in bones around the knee. About 80–90% of these tumors develop in the ends of the long bones that form the knee. The second most common site for these tumors is in the ends of the upper arm bone close to the shoulder; they can also be in other places, like the pelvis, shoulder and skull. Osteosarcoma usually occurs in teenagers, is twice as common in males and is diagnosed around 15 years of age ( 1 ) . Approximately 20% of children diagnosed with osteosarcoma have an advanced stage that has metastasized to the lungs, brain and other bones ( 2 ) . If metastases are present when the osteosarcoma is first diagnosed, the 5-year survival rate is about 15 to 30%. The survival rate is closer to 40% if the cancer has spread only to the lungs (as opposed to having reached other organs), or if all of the tumors (including metastases) can be removed with surgery ( 3 ) .

Pediatric soft tissue sarcomas (STSs) are a heterogeneous group of malignant tumors that originate from primitive mesenchymal tissue and account for 7% of all childhood tumors ( 4 ) . Rhabdomyosarcoma, a tumor of striated muscle, is the most common soft tissue sarcoma in children aged 0 to 14 years and accounts for 50% of tumors in this age group ( 4 ) . The remaining STSs are a heterogeneous group of tumors and include neoplasms of connective tissue (fibrous and adipose), peripheral nervous system, smooth muscle (leiomyosarcomas) and vascular tissue ( 4 ) . Though rhabdomyosarcoma can appear at various sites, it primarily presents in the head and neck (35%), the genitourinary tract (22%) and the extremities (18%) ( 5 ) . Of the two main histological types of pediatric rhabdomyosarcoma, embryonic and alveolar, embryonal is more prevalent, contributing to roughly 53% of all diagnosed cases; it generally presents in children under fifteen in either the head and neck regions or the genitourinary tract ( 6 ) . Alveolar rhabdomyosarcoma generally affects the muscles of the extremities or trunk and has been found to be more resistant to treatment and more likely to spread to regional lymph nodes than the embryonal type ( 7 ) . At diagnosis, roughly 50% of rhabdomyosarcoma cases consist of patients five and younger and 25% of all patients have metastatic disease ( 8 ) .

Metastasis occurs secondary to cancer cell detachment from the primary tumor, invasion through degraded basement membrane into the surrounding stroma, and entry into and transport through the vascular or lymphatic system to distal sites such as the liver, lungs and brain, and extravasation, tumor cell proliferation and angiogenesis at distal sites ( 9 – 13 ) . Tumor cell invasion depends upon degradation of the extra-cellular matrix (ECM), which, when intact, acts as a barrier to block cancer cell invasion. The ECM is composed of collagen, proteoglycans, fibronectin, laminin and other glycoproteins ( 14 – 16 ) . Two families of proteases, the matrix metalloproteinases (MMPs) and urokinase plasminogen activators (u-PA) are involved in tumor invasion and metastasis. Numerous clinical and experimental studies have demonstrated that elevated levels of u-PA and MMPs are associated with cancer progression, metastasis and shortened survival ( 17 – 20 ) .

MMPs, especially MMP-2 and MMP-9 play key roles in tumor cell invasion and metastasis due to their ability to degrade type IV collagen, a major component of the ECM ( 16 , 21 , 22 ) . MMP-2 and -9 are secreted as inactive pro-enzymes in their latent zymogenic form, and activated by other MMPs or proteases. Proteolytic activities of MMP-2 and MMP-9 are inhibited by specific inhibitors, tissue inhibitors of metalloproteinases (TIMPs). Thus, a critical determinant of net proteolytic degradation is the balance between MMP and TIMP levels. Clinical studies note the high levels of MMP-9 expression in the highly malignant bone tumor osteosarcoma ( 23 , 24 ) . Of patients with osteosarcoma treated with excision alone, 80% develop pulmonary metastases, which suggest that micrometastases are present at diagnosis ( 23 ) . Studies of soft tissue sarcomas also note correlation of metastasis and poor prognosis with elevated MMP-2 and or MMP-9 and lack of TIMP-2 expression ( 19 ) .

The serine protease u-PA converts plasminogen to plasmin, which is capable of promoting tumor growth and angiogenesis, degrading the ECM and basement membrane and activating pro-MMPs ( 25 ) . Components of the u-PA system such as u-PA, plasminogen activator inhibitor-1 (PAI-1), and urokinase-type plasminogen activator receptor (u-PAR) are overexpressed in a variety of cancer types, most notably in breast cancer ( 26 ) , but also in sarcomas ( 18 ) , and correlate with cancer progression, metastasis and poor prognosis. Thus the u-PA system represents a potential target for anticancer strategies.

Rath and Pauling ( 27 ) proposed using nutrients such as lysine and ascorbic acid to target plasmin-mediated connective tissue degradation as a universal approach to tumor growth and expansion. Binding to plasminogen active sites, lysine blocks plasminogen activation into plasmin by tissue plasminogen activator (t-PA). Thus it modulates the plasmin-induced MMP activation cascade ( 28 ) . Subsequent studies confirmed this approach and led to identifying a novel formulation composed of lysine, ascorbic acid, proline and green tea extract and other micronutrients (NM), which has shown significant anticancer activity against a large number (∼40) of cancer cell lines, blocking cancer growth, tissue invasion and MMP expression both *in vitro* and *in vivo* ( 29 ) . In this study, we focused on the modulating effect of NM on the activities of MMP-2 and -9, TIMPs and u-PA in pediatric human sarcomas: osteosarcoma and rhabdomyosarcoma cell lines.

## Materials and methods

### Materials

Human pediatric sarcoma cell lines osteosarcoma MNNG-HOS, osteosarcoma U-2OS and embryonal rhabdomyosarcoma RD, along with their culture media were obtained from ATCC. Antibiotics, penicillin and fetal bovine serum (FBS), were obtained from Gibco-BRL (Long Island, NY). Twenty-four-well tissue culture plates were obtained from Costar (Cambrdige, MA). Gelatinase zymography was performed in 10% Novex pre-cast SDS polyacrylamide gel (Invitrogen Inc.) with 0.1% gelatin in non-reducing conditions. The nutrient mixture (NM), prepared by VitaTech (Hayward, CA) was composed of the following ingredients in the relative amounts indicated: vitamin C (as ascorbic acid and as Mg, Ca and palmitate ascorbate) 700 mg; L-lysine 1,000 mg; L-proline 750 mg; L-arginine 500 mg; N-acetyl cysteine 200 mg; standardized green tea extract (80% polyphenol) 1,000 mg; selenium 30 *μ* g; copper 2 mg; manganese 1 mg. All other reagents used were of high quality and were obtained from Sigma, unless otherwise indicated.

### Cell cultures

The sarcoma cell lines were grown in their respective media: osteosarcoma MNNG-HOS in MEM, osteosarcoma U-2OS in McCoy medium, and rhabdomyosarcoma in DME, supplemented with 10% FBS, penicillin (100 U/ml), and streptomycin (100 *μ* g/ml) in 24-well tissue culture plates. The cells were plated at a density of 1×10 ^5^ cells/ml and grown to confluency in a humidified atmosphere at 5% CO _2_ at 37°C. Serum-supplemented media were removed and the cell monolayer was washed once with PBS with the recommended serum-free media. The cells were treated with the nutrient mixture, dissolved in media and tested at 0, 50, 100, 250, 500 and 1,000 *μ* g/ml in triplicate at each dose for u-PA and TIMP-2 studies. For MMP analysis, cells were treated with NM at 0, 10, 50, 100, 500 and 1,000 *μ* g/ml. Parallel sets of cultures were treated with PMA (100 ng/ml) for induction of MMP-9. Control and PMA treatments were done in triplicates. The plates were then returned to the incubator. The conditioned media were collected separately, pooled, and centrifuged at 40°C for 10 min at 3,000 rpm to remove cells and cell debris. The supernatant was collected and used to assess for u-PA activity (by fibrin zymography on 10% SDS-PAGE gels containing fibrinogen and plasminogen), MMP-2 and -9 (by gelatinase zymography), and TIMPs (by reverse zymography).

### Fibrin zymography

Fibrin zymography was used to analyze u-PA activity on 10% SDS-PAGE gels containing fibrinogen (5.5 mg/ml) and plasminogen (50 *μ* g/ml). After electrophoresis, the gels were washed twice with 2.5% Triton X-100 for 30 min. The gels were then incubated overnight at 37°C with 0.1% glycine buffer pH 7.5 and then stained with 0.5% Coomassie Brilliant Blue R250 and destained. Electrophoresis of u-PA and t-PA were conducted for comparison. Fibrin zymograms were scanned using CanoScan 9950F Canon Scanner.

### Gelatinase zymography

Gelatinase zymography was performed in 10% NOVEX Pre-Cast SDS Polyacrylamide Gel (Invitrogen Corporation) in the presence of 0.1% gelatin under non-reducing conditions. Culture media (20 *μ* l) were mixed with sample buffer and loaded for SDS-PAGE with tris glycine SDS buffer as suggested by the manufacturer (Novex). Samples were not boiled before electrophoresis. Following electrophoresis the gels were washed twice in 2.5% Triton X-100 for 30 min at room temperature to remove SDS. The gels were then incubated at 37°C overnight in substrate buffer containing 50 mM Tris-HCl and 10 mM CaCl _2_ at pH 8.0 and stained with 0.5% Coomassie Blue R250 in 50% methanol and 10% glacial acetic acid for 30 min and destained. Upon renaturation of the enzyme, the gelatinases digest the gelatin in the gel and give clear bands against an intensely stained background. Protein standards were run concurrently and approximate molecular weights were determined by plotting the relative mobilities of known proteins.

### Reverse zymography

TIMPs were analyzed by reverse zymography on 15% SDS gels containing serum-free conditioned medium from cells. After electrophoresis the gels were washed twice with 2.5% Triton X-100 for 30 min at room temperature to remove SDS. The gels were then incubated at 37°C overnight in 50 mM Tris-HCl and 10 mM CaCl _2_ at pH 7.6 and stained with 0.5% Coomassie Blue R25, destained and scanned.

### Scanning of gelatinase, reverse and fibrin zymograms

Gelatinase, reverse and fibrin zymograms were scanned using CanoScan 9950F Canon scanner at 300 dpi. The intensity of the bands was evaluated using the pixel-based densitometer program Un-Scan-It, Version 5.1, 32-bit, by Silk Scientific Corporation (Orem, UT, USA), at a resolution of 1 Scanner Unit (1/100 of an inch for an image that was scanned at 100 dpi). The pixel densitometer calculates the optical density of each pixel (values 0 to 255) using the darkly stained background of the gel as a pixel value of 0. A logarithmic optical density scale was used since the optical density of film and gels is logarithmically proportional to the concentration. The pixel densitometer sums the optical density of each pixel to give the band density.

### Statistical analysis

Pearson;s correlation coefficient was determined between NM effect on mean MMP-2 or MMP-9, u-PA and TIMP-2 expressions of sarcoma cell lines using MedCalc Software (Mariakerke, Belgium).

## Results

Table I provides an overview of the tested pediatric sarcoma cell line u-PA, MMP and TIMP-2 activities.

### Effect of NM on u-PA activity in human pediatric sarcoma cell lines

**Figure 1.**
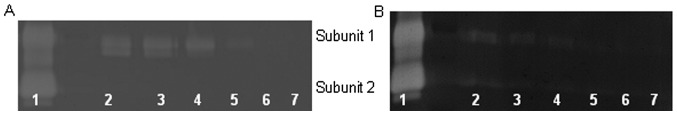

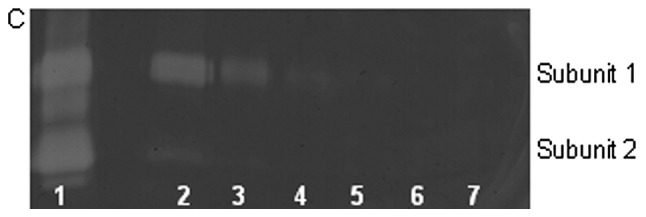

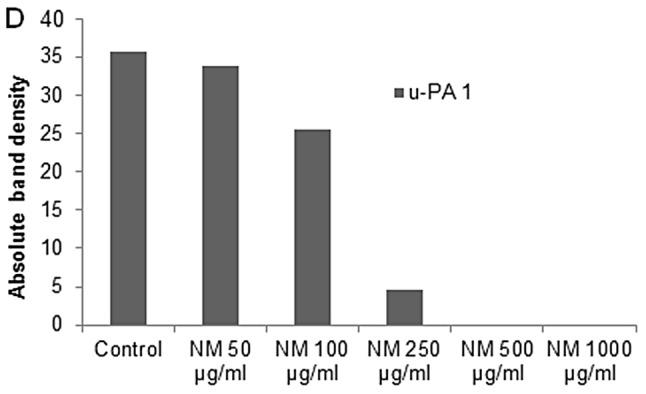

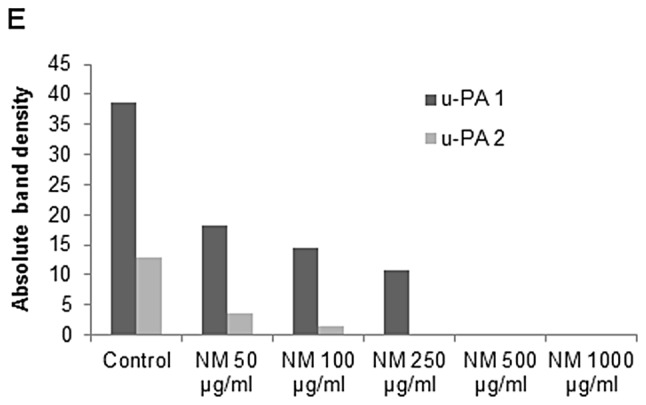

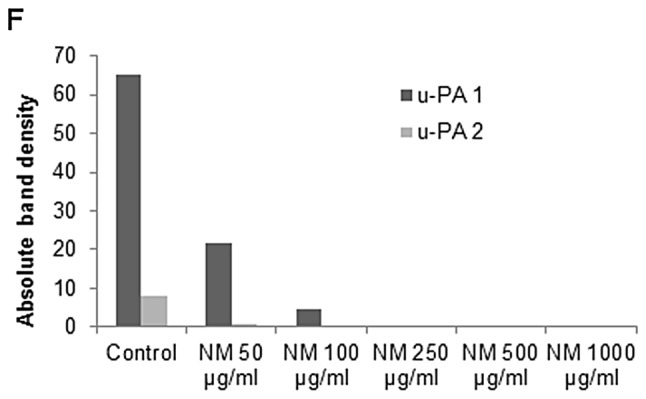
Effect of NM on osteosarcoma MNNG-HOS, osteosarcoma U-2OS and rhabdomyosarcoma RD u-PA expression. Fibrin zymograms of (A) MNNG-HOS, (B) U-2OS, and (C) RD u-PA expression. Legend: 1, u-PA; 2, markers; 3, control; 4–8, NM 50, 100, 250, 500 and 1,000 *μ* g/ml, respectively. Densitometric analyses of (D) MNNG-HOS, (E) U-2OS and (F) RD u-PA expression.

Activity of u-PA was detected in osteosarcoma MNNG-HOS and U-2OS and rhabdomyosarcoma RD cell lines. MNNG-HOS showed one band corresponding to subunit 1 (55 kD) and U-2OS and RD showed bands corresponding to subunits 1 and 2 at 55 and 33 kD. NM exerted dose response inhibition with virtual block of u-PA 1 activity at 500 *μ* g/ml in MNNG-HOS (linear trend R ^2^ =0.899) and 500 *μ* g/ml (linear trend R ^2^ =0.878) for u-PA-1 and 250 *μ* g/ml (linear trend R ^2^ =0.658) u-PA-2 in U-2OS. NM inhibited u-PA subunits 1 and 2 in RD dose-dependently with virtual block of u-PA-1 at 250 *μ* g/ml and u-PA-2 at 50 *μ* g/ml (linear trends R ^2^ =0.667 and 0.493 for subunits 1 and 2, respectively). See Fig. 1 for respective fibrin zymograms and densitometry analyses.

### Effect of NM on MMP-2 and MMP-9 expression by osteosarcoma cell line MNNG-HOS

**Figure 2.**
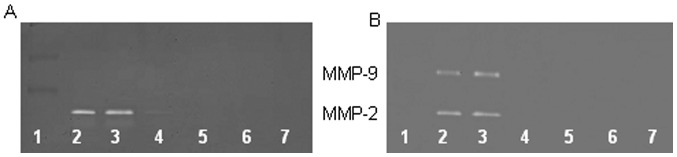

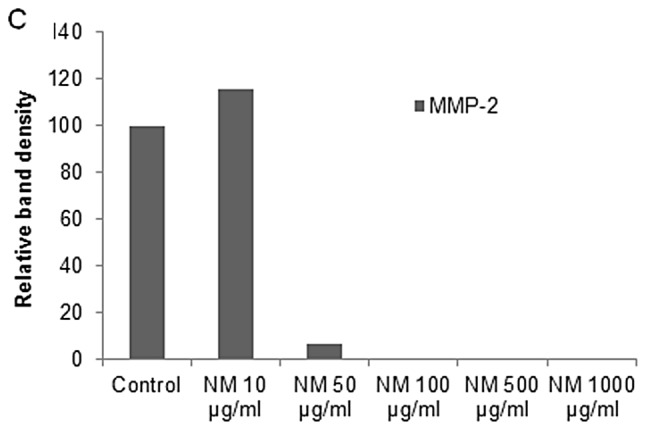

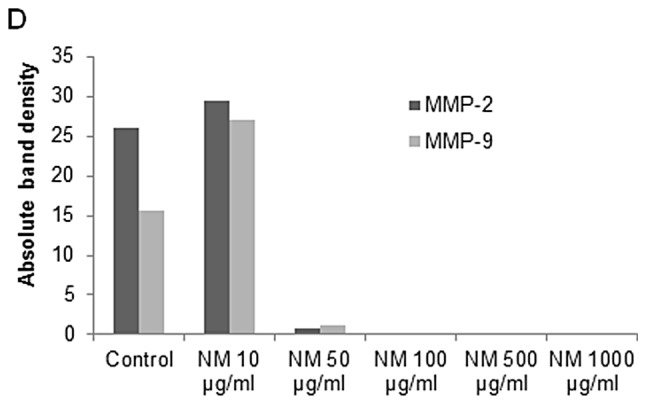
Effect of NM on osteosarcoma MNNG-HOS MMP-2 and -9 expression. Gelatinase zymograms of (A) normal and (B) PMA (100 ng/ml)-treated MNNG-HOS cell MMP-2 and MMP-9 expression. Legend: 1, markers; 2, control; 3–7, NM 10, 50, 100, 500 and 1,000 *μ* g/ml, respectively. Densitometric analyses of (C) normal and (D) PMA-treated MNNG-HOS cell MMP-2 and -9 secretion.

On gelatinase zymography, MNNG-HOS cells demonstrated strong expression of MMP-2 and induced MMP-9 with PMA (100 ng/ml) treatment that were inhibited by NM in a dose-dependent fashion with virtual total inhibition of MMP-2 and MMP-9 at 100 *μ* g/ml (linear trends R ^2^ =0.675 and 0.559, respectively) See Fig. 2 for gelatinase zymograms and densitometry analyses.

### Effect of NM on MMP-2 and MMP-9 expression by osteosarcoma cell line U-2OS

**Figure 3.**
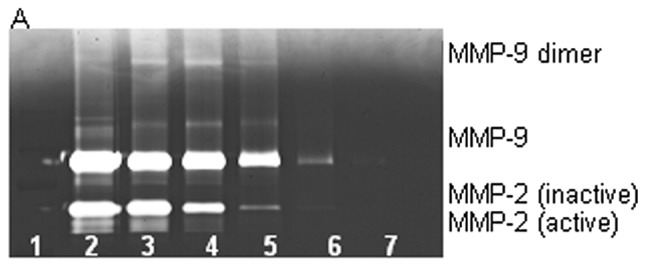

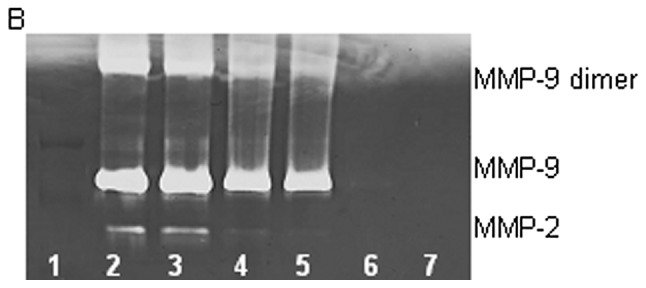

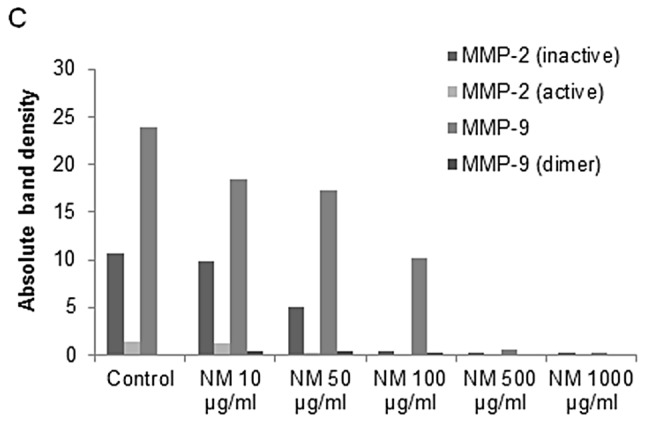

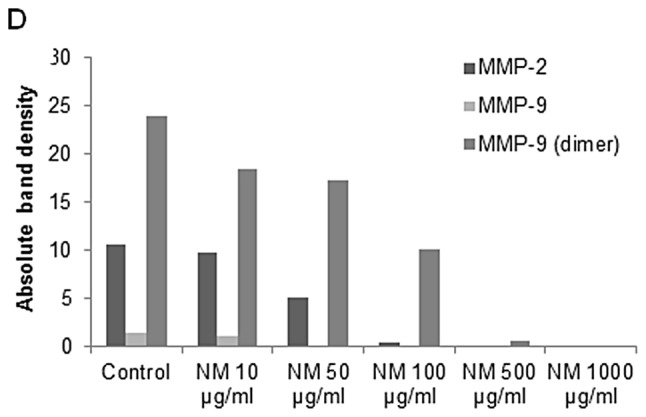
Effect of NM on osteosarcoma U-2OS MMP-2 and -9 expression. Gelatinase zymograms of (A) normal and (B) PMA (100 ng/ml)-treated U-2OS cell MMP-2 and MMP-9 expression. Legend: 1, markers; 2, control; 3–7, NM 10, 50, 100, 500 and 1,000 *μ* g/ml, respectively. Densitometric analyses of (C) normal and (D) PMA-treated U-2OS cell MMP-2 and -9 secretion.

On gelatinase zymography, U-2OS cells demonstrated strong expression of MMP-9 and slight expression of MMP-2 with and without PMA (100 ng/ml) treatment that were inhibited by NM in a dose-dependent fashion with virtual total inhibition of MMP-2 at 500 *μ* g/ml (linear trend R ^2^ =0.824) and MMP-9 at 1,000 *μ* g/ml (linear trend R ^2^ =0.816). See Fig. 3 for gelatinase zymograms and densitometry analyses.

### Effect of NM on MMP-2 and MMP-9 expression by rhabdomyosarcoma cell line RD

**Figure 4.**
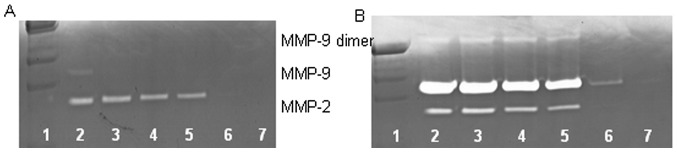

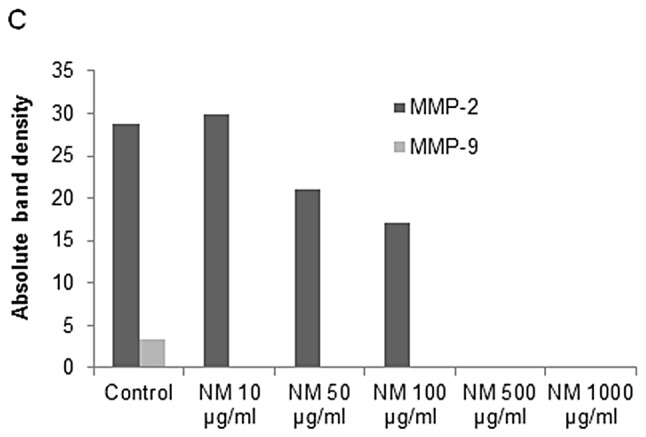

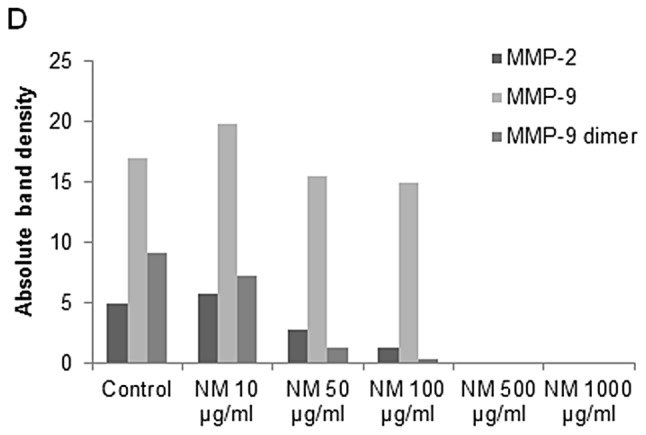
Effect of NM on rhabdomyosarcoma RD MMP-2 and -9 expression. Gelatinase zymograms of (A) normal and (B) PMA (100 ng/ml)-treated RD MMP-2 and MMP-9 expression. Legend: 1, markers; 2, control; 3–7, NM 10, 50, 100, 500 and 1,000 *μ* g/ml, respectively. Densitometric analyses of (C) normal and (D) PMA-treated RD MMP-2 and -9 secretion.

Zymography demonstrated strong expression of MMP-2 and slight expression of MMP-9 by RD cells that were inhibited by NM in a dose-dependent fashion with virtual total inhibition of MMP-2 at 500 *μ* g/ml (linear trend R ^2^ =0.899) and MMP-9 at 10 *μ* g/ml (linear trend R ^2^ =0.429). PMA (100 ng/ml) treatment profoundly enhanced MMP-9 expression and MMP-9 dimer by RD cells and decreased MMP-2 expression; NM inhibited MMP-2 and MMP-9 in a dose-dependent manner with total block of MMP-2 and MMP-9 and MMP-9 dimer at 500 *μ* g/ml (linear trends R ^2^ =0.876, 0.769 and 0.800, respectively). See Fig. 4 for gelatinase zymograms and densitometry analyses.

### Effect of NM on TIMP activity in osteosarcoma MNNG-HOS and U-2OS and rhabdomyosarcoma RD

**Figure 5.**
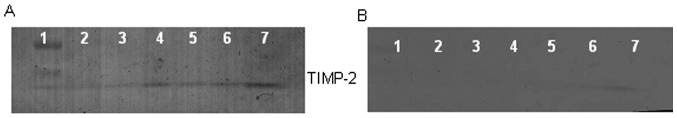

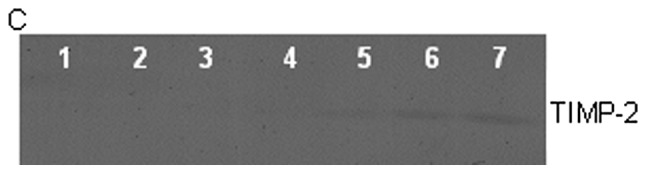

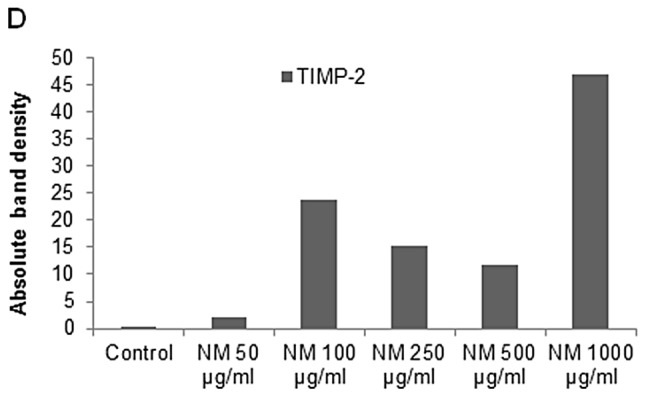

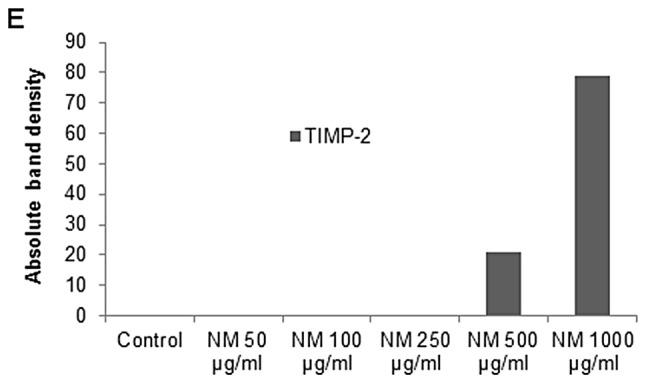

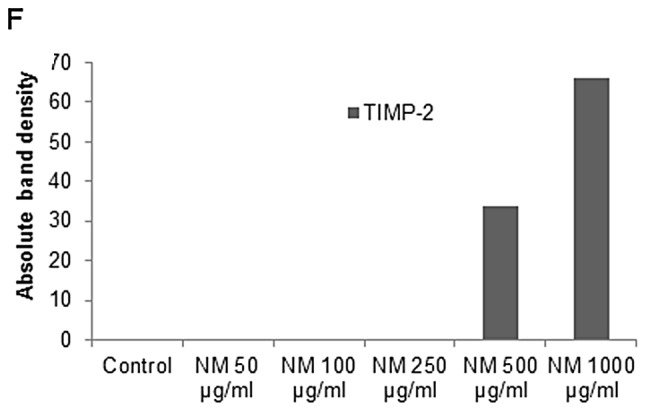
Effect of NM on osteosarcoma MNNG-HOS, osteosarcoma U-2OS and rhabdomyosarcoma RD TIMP-2 expression. Reverse zymograms of (A) MNNG-HOS, (B) U-2OS and (C) RD TIMP-2 expression. Legend: 1, markers; 2, control; 3–7, NM 50, 100, 250, 500 and 1,000 *μ* g/ml, respectively. Densitometric analyses of (D) MNNG-HOS, (E) U-2OS and (F) RD TIMP-2 expression.

Reverse zymography revealed upregulation of TIMP-2 activity with NM treatment in all cancer cell lines in a dose-dependent manner. Minimum activity was expressed at 50 and maximum at 1,000 *μ* g/ml NM. See Fig. 5 for respective reverse zymograms and densitometry analyses.

### Correlation between pediatric sarcoma u-PA, TIMP-2 and MMP expressions

**Figure 6.**
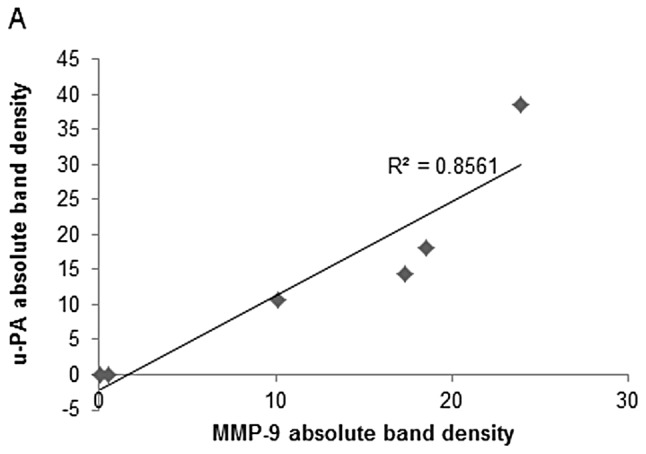

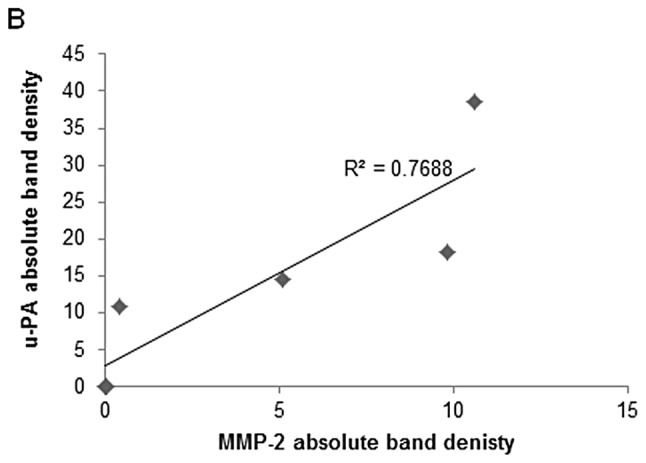

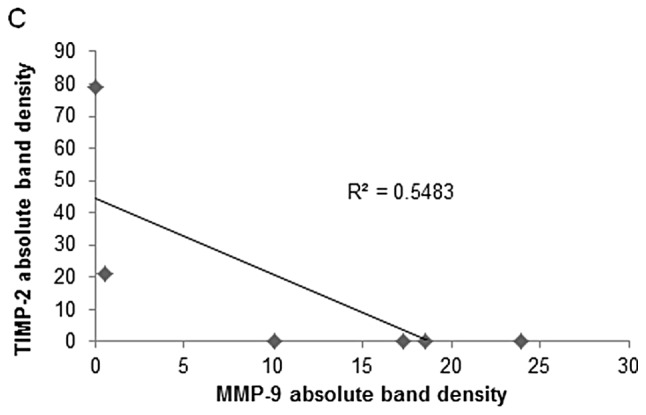

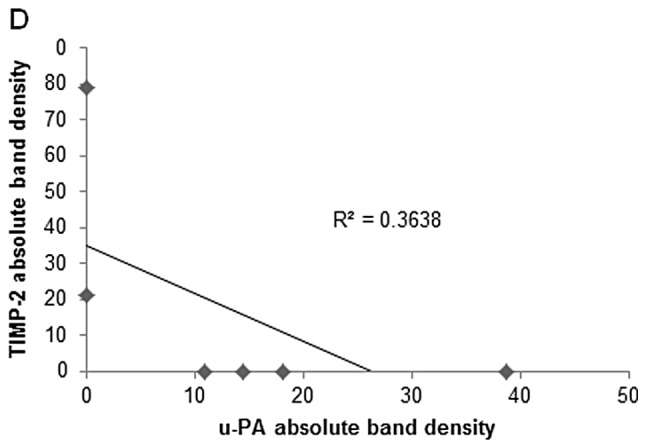
(A) Correlation between the effects of NM on osteosarcoma U-2OS u-PA and MMP-9 expression (correlation coefficient r= 0.925). (B) Correlation between the effects of NM on osteosarcoma U-2OS MMP-2 and u-PA expression (correlation coefficient r= 0.877). (C) Correlation between the effects of NM on osteosarcoma U-2OS TIMP-2 and MMP-9 expression (correlation coefficient r=−0.740). (D) Correlation between the effects of NM on osteosarcoma U-2OS TIMP-2 and u-PA expression (correlation coefficient r=−0.603).

Analysis revealed a positive correlation between u-PA and MMP-2 expressions of NM-treated osteosarcoma MNNG-HOS and between u-PA and MMP-2 and MMP-9 expressions of osteosarcoma U-2OS and rhabdomyosarcoma RD, as shown in Table II . Fig. 6A shows the correlation graph for osteosarcoma U-2OS u-PA and MMP-2/MMP-9 with correlation coefficients r=0.925 (MMP-9) and 0.877 (MMP-2). Negative correlations were found between the expressions of MMP-2 or MMP-9 and TIMP-2 in all pediatric sarcoma cell lines treated with NM that secreted TIMP-2, as shown in Table II . The correlation (r=−0.740) between MMP-9 and TIMP-2 is shown for osteosarcoma U-2OS in Fig. 6B . Negative correlations were found between expressions of TIMP-2 and u-PA in all NM-treated sarcoma cell lines studied. The correlation (r=−0.603) between u-PA and TIMP-2 is shown for osteosarcoma U-2OS in Fig. 6C .

## Discussion

Two families of proteases, urokinase plasminogen activators and matrix metalloproteinases, play key roles in tumor cell invasion and metastasis. Urokinase plasminogen has a demonstrated role as an initiator of ECM proteolysis and associated tumor cell invasion ( 28 ) . The protease u-PA cleaves plasminogen to plasmin, which is capable of promoting tumor growth and angiogenesis, degrading the ECM and basement membrane and activating pro-MMPs ( 25 , 30 ) . The uPA-uPAR system is a key regulator of osteosarcoma invasion and an inverse relationship has been demonstrated between u-PA levels and survival time ( 18 , 30 ) . Overexpression of u-PA in soft-tissue sarcoma patients has also been correlated with cancer progression, metastasis and poor prognosis ( 20 ) . Matrix metalloproteinases, especially MMP-2 and MMP-9, are also key regulators of tumor cell invasion and metastasis due to their ability to degrade type IV collagen, a major component of the ECM. Overproduction of MMPs, especially MMP-2 and -9 and low levels of TIMPs have been shown to be associated with a more aggressive behavior of sarcomas ( 19 , 23 , 31 , 32 ) . For example, increased expression of MMP-9 has been found to correlate with osteosarcoma metastasis in patients and inhibitors of MMPs, such as TIMP-1 have been shown to inhibit invasiveness of osteosarcoma tumor cells *in vitro* ( 23 , 24 , 31 ) . A study of the immunohistochemical expression of MMPs and TIMPS in human rhabdomyosarcoma demonstrated strong MMP-1, -3 and -9 expression in rhabdomyosarcoma, alveolar RMS greater than embryonal RMS. Intratumor vessels and perivascular ECM were positive for MMP-9 and negative for TIMPS in both types ( 33 ) .

Our study demonstrated that the specific mixture of nutrients tested significantly inhibited u-PA secretion in osteosarcoma MNNG-HOS and U-2OS and in rhabdomyosarcoma RD cell lines. Furthermore, the NM demonstrated dose-dependent decrease in MMP secretion and increase in TIMP-2 secretion by all sarcoma cell lines. As expected, a significant positive correlation was found between the secretion of u-PA and MMP-2/MMP-9 and a significant negative correlation between u-PA and TIMP-2 and between MMP-2/MMP-9 and TIMP-2 secretion by NM treatment of osteosarcoma and rhabdomyosarcoma cells. Furthermore, a previous study demonstrated significant correlation between NM inhibition of Matrigel invasion and NM modulation of the MMP-2 and -9 activities of the sarcoma cell lines studied ( 34 ) . A significant negative correlation was found between NM modulation of Matrigel invasion inhibition and MMP-2 secretion with osteosarcoma MNNG-HOS (r=−0.6531), osteosarcoma U-2OS (r=−0.835) and rhabdomyosarcoma RD (r=−0.675). A previous *in vivo* study of the effects of NM 0.5% on xenograft tumor growth of osteosarcoma MNNG-HOS cells in nude mice support these results in that it demonstrated significant inhibition of xenograft tumor growth (53%, p=0.0001), tumor vascularity and VEGF and MMP-9 tumor tissue staining compared to tumors of mice in the control diet group ( 35 ) .

The standard treatments for sarcomas include surgery, chemotherapy and radiation with severe associated toxicity and limited efficacy, clearly indicating a need for safe and effective therapeutic approaches to control progression and metastasis, especially in these young sarcoma patients. Extensive research documents the efficacy and safety of dietary and botanical natural compounds in cancer prevention ( 36 ) . The nutrient mixture was formulated by selecting nutrients that act on critical physiological targets in cancer progression and metastasis, as documented in both clinical and experimental studies. Combining these micronutrients expands metabolic targets, maximizing biological impact with lower doses of components. A previous study of the comparative effects of NM, green tea extract and EGCG on inhibition of MMP-2 and MMP-9 secretion of different cancer cell lines with varying MMP secretion patterns, revealed the superior potency of NM over GTE and EGCG at equivalent doses ( 37 ) . These results can be understood from the more comprehensive treatment offered by the combination of nutrients in NM over individual components of NM since MMP-2 and MMP-9 are mediated by differential pathways.

Optimal ECM structure depends upon adequate supplies of ascorbic acid and the amino acids lysine and proline to ensure proper synthesis and hydroxylation of collagen fibers. In addition, lysine contributes to ECM stability as a natural inhibitor of plasmin-induced proteolysis ( 27 , 38 ) . Manganese and copper are also essential for collagen formation. There is considerable documentation of the potency of green tea extract in modulating cancer cell growth, metastasis, angiogenesis, and other aspects of cancer progression ( 39 – 45 ) . N-acetyl cysteine and selenium have demonstrated inhibition of tumor cell MMP-9 and invasive activities, as well as migration of endothelial cells through ECM ( 46 – 48 ) . Ascorbic acid demonstrates cytotoxic and antimetastatic actions on malignant cell lines ( 49 – 53 ) and cancer patients have been found to have low levels of ascorbic acid ( 54 , 55 ) . Low levels of arginine, a precursor of nitric oxide (NO), can limit the production of NO, which has been shown to predominantly act as an inducer of apoptosis ( 56 ) .

In conclusion, the NM demonstrated potent anticancer activity by targeting primary mechanisms responsible for the aggressive spread of pediatric sarcomas. In this *in vitro* study, the NM significantly inhibited secretion of u-PA and MMP-2 and/or -9 and increased secretion of TIMP-2 in osteosarcoma and rhabdomyosarcoma cells, suggesting its potential in modulating cancer invasion and metastasis. NM inhibition of MMP secretion was found to be correlated significantly with Matrigel invasion of all the sarcoma cell lines studied ( 34 ) . Furthermore, use of the nutrient mixture would not pose any toxic effect clinically, especially in the relevant doses, as *in vivo* safety studies demonstrate. An *in vivo* toxicology study (Roomi *et al* , J AM Coll Nutr 22: abs. 86, 2003) showed that NM had no adverse effects on vital organs (heart, liver and kidney), or on the associated functional serum enzymes.

## Figures and Tables

**Table I.** Overview of MMP-2, MMP-9, u-PA and TIMP-2 expression of pediatric sarcoma cell lines.

Cancer cell line	MMP-2	MMP-9	u-PA	TIMP-2
Osteosarcoma MNNG-HOS	+	With PMA	+	+
Osteosarcoma U-2OS	+	+	+	+
Rhabdomyosarcoma RD	+	+	+	+

**Table II.** Correlation between effects of NM on sarcoma cell u-PA, MMPs and TIMPs.

Cell line	u-PA and MMPs	MMPs and TIMPs	u-PA and TIMPs
Osteosarcoma MNNG-HOS	r=0.845 (MMP-2)	r=−0.696 (MMP-2)	r=−0.623
Osteosarcoma U-2OS	r=0.925 (MMP-9)	r=−0.740 (MMP-9)	r=−0.603
	r=0.877 (MMP-2)		
Rhabdomyosarcoma RD	r=0.511 (MMP-9)	r=−0.868 (MMP-2)	r=−0.423
	r=0.660 (MMP-2)		
